# A Novel Experimental Study on the Rheological Properties and Thermal Conductivity of Halloysite Nanofluids

**DOI:** 10.3390/nano10091834

**Published:** 2020-09-14

**Authors:** Thong Le Ba, Ahmed Qani Alkurdi, István Endre Lukács, János Molnár, Somchai Wongwises, Gyula Gróf, Imre Miklós Szilágyi

**Affiliations:** 1 Department of Inorganic and Analytical Chemistry, Budapest University of Technology and Economics, Muegyetem rakpart 3, 1111 Budapest, Hungary; ahmed.qani@yahoo.com (A.Q.A.); imre.szilagyi@mail.bme.hu (I.M.S.); 2 Institute for Technical Physics and Materials Science, Centre for Energy Research, Hungarian Academy of Sciences, Konkoly Thege M. út 29-33, 1121 Budapest, Hungary; lukacs.istvan@energia.mta.hu; 3 Department of Physical Chemistry and Materials Science, Budapest University of Technology and Economics, Muegyetem rakpart 3., 1111 Budapest, Hungary; molnar.janos@mail.bme.hu; 4 Department of Mechanical Engineering, Faculty of Engineering, King Mongkut’s University of Technology Thonburi (KMUTT), Bangmod, Bangkok 10140, Thailand; somchai.won@kmutt.ac.th; 5 National Science and Technology Development Agency (NSTDA), Pathum Thani 12120, Thailand; 6 Department of Energy Engineering, Faculty of Mechanical Engineering, Budapest University of Technology and Economics, Műegyetem rkp. 3, 1111 Budapest, Hungary; grof@energia.bme.hu

**Keywords:** halloysite, surfactant, nanofluids, thermal conductivity, viscosity

## Abstract

Nanofluids obtained from halloysite and de-ionized water (DI) were prepared by using surfactants and changing pH for heat-transfer applications. The halloysite nanotubes (HNTs) nanofluids were studied for several volume fractions (0.5, 1.0, and 1.5 vol%) and temperatures (20, 30, 40, 50, and 60 °C). The properties of HNTs were studied with a scanning electron microscope (SEM), energy-dispersive X-ray analysis (EDX), Fourier-transform infrared (FT-IR) spectroscopy, X-ray powder diffraction (XRD), Raman spectroscopy and thermogravimetry/differential thermal analysis (TG/DTA). The stability of the nanofluids was proven by zeta potentials measurements and visual observation. With surfactants, the HNT nanofluids had the highest thermal conductivity increment of 18.30% for 1.5 vol% concentration in comparison with the base fluid. The thermal conductivity enhancement of nanofluids containing surfactant was slightly higher than nanofluids with pH = 12. The prepared nanofluids were Newtonian. The viscosity enhancements of the nanofluid were 11% and 12.8% at 30 °C for 0.5% volume concentration with surfactants and at pH = 12, respectively. Empirical correlations of viscosity and thermal conductivity for these nanofluids were proposed for practical applications.

## 1. Introduction

Conventional heat-transfer fluids such as water, propylene glycol, ethylene glycol, and engine oil have been broadly utilized in many industrial applications. The heat-transfer enhancement of these fluids can reduce the material cost, energy, process time, and size, and increase the lifetime of the device [1,2].

In the heat-exchange systems, one of the problems is that the conventional heat-transfer fluids have low thermal conductivity. The thermal conductivity of these fluids can be enhanced by dispersing the solid particles. The study of the thermal conductivity of mixtures of solid particles and liquids was first developed in the 19th century when James Clerk Maxwell dispersed small particles into liquids. Further studies were conducted with millimeter, micro-sized particles. These particles enhance the properties of fluids. However, the major problem with these particles is that they settle rapidly in the fluids. Additionally, this causes a pressure drop and erosion of pipelines. The issues may be resolved by using nano-sized particles [3].

Nano-suspensions are the new class of nanotechnology-based heat-transfer fluid. First, aluminum oxide (Al_2_O_3_) ultrafine particles were dispersed into water by Masuda et al., and the thermal conductivity enhancement was 30% [4]. Then, the nanofluids were first introduced in 1995 by Choi et al. [5]. Since Choi introduced the concept of nanofluids, more researchers have started to search, develop and publish many articles about them. From 1993 to 2019 only, more than 11,000 articles were published and in 2019 the number of the published articles was 2005 [6].

Generally, nanofluids consist of two main parts: the nanoparticles and the base fluid. Nanofluids can be prepared from many different combinations, examples of the solid particles are metal- (metals: Al, Cu, Ag, etc.; metal oxides: Al_2_O_3_, CuO, TiO_2_, etc.; metal carbides: TiC), metalloid- (SiC, SiO_2_) and non-metal (carbon materials: graphite, diamond, graphene, etc.) based nanomaterials. Examples for the base fluids are water, ethylene glycol, ethanol, oil, and other conventional fluids. Nanoparticles are used to enhance the useful properties of liquids, modify their rheological behavior [6]. In the nanofluids, the nanoparticles have a complicated movement with coagulation, thermophoresis effect and Brownian motion. These factors depend on the concentration, temperature, size, shape, and type of nanoparticles, and so on. In the literature, it was shown that these factors play an important role in increasing the thermal conductivity and viscosity [7,8,9,10].

In 1999, Lee et al. [11] used Al_2_O_3_ and CuO nanoparticles in water and ethylene glycol. They found that thermal conductivity was linearly dependent on the volume fraction. In 2001, Eastman et al. [12] dispersed nanometer-sized copper particles in ethylene glycol, and the effective thermal conductivity was much higher than the base fluid. Choi et al. showed that non-metallic nanomaterials, multiwall carbon nanotubes (CNTs), in water increased the thermal conductivity up to 160% at 1% volume fraction. After that, much research on heat-transfer fluids was performed with different nanoparticles, such as aluminum [13,14,15], gold [16], copper oxide [17], CNT [18,19], silicon dioxide, titanium dioxide [20].

Nonetheless, the results from various research groups were different for the same materials. This can be explained by preparation techniques and the agglomeration state in nanofluids. Buongiorno et al. [3] performed benchmark research to compare the results of thermal conductivity obtained by different investigators. The same samples were measured in different locations, and with different methods, then the results and measurement error could be evaluated.

There is a lack of agreement between theory and experimental results. Some heat-transfer mechanisms have been proposed, such as liquid-layering, aggregation, particle motion, etc. [1]. In the aggregation mechanism, thermal conductivity occurs along with large particles or aggregates. This means that the size and shape of particles and clusters play an important role in thermal conductivity enhancement [21,22,23]. Because it was found that materials with chain-like structures, nanofibers or nanotubes have the highest thermal conductivity, much research has been performed on applications of CNTs nanofluids [24,25,26,27], titanium dioxide nanotube [28], titanate nanotube [29], halloysite nanotube nanofluids [30], etc. Venerus et al. [31] implemented the benchmark research for the comparison of viscosity values of the same samples from different research groups.

The use of nanoparticles improves the thermal conductivity of fluids and increases their viscosity, which causes an increase in pump energy. This limits the industrial application of nanofluids in heat-transfer systems. Like thermal conductivity, the viscosity of nanofluids also depends on the size and shape of particles and clusters. Therefore, the combined investigation of viscosity and thermal conductivity is essential. Many studies, including on both of these issues, have been performed [4,32,33,34].

Halloysite, belonging to the kaolin group, is a low-cost nanotubular clay with the chemical formula Al_2_Si_2_O_5_(OH)_4_·nH_2_O, where *n* = 0–2 [35]. The length of the halloysite is from 0.02 to 30 µm, the inner diameter is from 10 to 100 nm, and the outer diameter is approximately 30 to 190 nm [36,37]. The inner surface includes Al–OH groups, while the outer surface comprises inert Si–O–Si groups. Therefore, the reactivities of the outer and inner surfaces are different [38]. Because of these properties, there are many different applications of halloysite, such as solvent-free nanofluids [39,40], nanoreactors [41], drug delivery [42], energy storage devices, etc. [43]. However, the ability to apply halloysite as nanomaterials to prepare water-based nanofluids has rarely been investigated. Alberola et al. prepared the halloysite nanofluid and improved its stability by setting pH = 12. The studied temperature was from 40 to 80 °C. The thermal conductivity enhancement was 8% at 5% volume concentration and T = 80 °C, while the viscosity increased with halloysite content [30].

Usage of high pH for stabilization limits the applications of halloysite-based nanofluids. In this research, the halloysite-based nanofluid was investigated by dispersing halloysite into the water, and the stability was improved by different surfactants. As far as authors know, there are no studies on stabilizing halloysite nanofluids with surfactants and investigations on heat-transfer applications. In addition, the size of halloysite used in this research is smaller than in previous research. The halloysite nanotube (HNT) was first analyzed by scanning electron microscopy, Fourier-transform infrared spectroscopy, X-ray powder diffraction, energy-dispersive X-ray analysis, and thermogravimetric analysis. The concentrations of nanofluids were prepared from 0.5 to 1.5 vol%. The thermal conductivity and dynamic viscosity of these nanofluids were measured. The temperatures during the experiments are from 20 to 60 °C. In order to evaluate the measurement results, the nanofluids were prepared with pH = 12 and the same concentrations.

## 2. Materials and Methods

### 2.1. Materials

HNTs were supplied by the University of Pannonia. The surfactants including Tween, oleylamine, Gum Arabic (GA), hexadecyltrimethylammonium bromide (CTAB), sodium dodecyl sulfate (SDS) and sodium carboxymethylcellulose (SCMC) were bought from Sigma-Aldrich (Saint Louis, MO, USA). De-ionized water (DI) was used as base fluids. DI and 1M sodium hydroxide (NaOH) solution were supplied by the Department of Inorganic and Analytical Chemistry laboratory, Budapest University of Technology and Economics (Budapest, Hungary).

### 2.2. Preparation of Nanofluids

Halloysite nanofluids were prepared by dispersing different amounts of halloysite nanoparticles in DI base fluid. The volume concentrations of halloysite content were 0.5%, 1.0%, and 1.5%. Then, surfactants or 1 M NaOH solution were added to the nanofluids in the appropriate amount. Halloysite nanofluids were sonicated at 130 W and 45 kHz using an ultrasonication instrument for 1h. Table 1. shows pure halloysite nanofluid sample specifications.

### 2.3. Characterization Techniques

The halloysite powder samples were used, so the morphological characterization of halloysite was performed by a LEO 1440 XB scanning electron microscope (LEOGmbH, Oberkochen, Germany) at 5 kV with a secondary electron detector in a high vacuum mode.

The halloysite’s chemical components were investigated by using energy-dispersive X-ray analysis with a JEOL JSM-5500LV electron microscope (Tokyo, Japan). The crystal structure of the halloysite was studied by utilizing a X’PERT PRO MPD X-ray diffractometer (PANalytical, Almelo, Netherlands), with Cu K_α_ irradiation. The measurement results were recorded at a resolution of 3 degrees/min for the 2θ range of 5° to 65°. Fourier transform infrared (FT-IR) spectra of halloysite were investigated by an Excalibur FTS 3000 BioRad FT-IR (Bio-Rad, Digilab, UK) in the 400–4000 cm^−1^ domain in transmittance mode, with a resolution of 4 cm^−1^, and the number of scans was 64. Raman spectrum was obtained utilizing a Jobin Yvon Labram Raman spectrometer (Horiba, Kyoto, Japan) containing an Olympus BX41 microscope (Olympus, Tokyo, Japan) equipped with a green Nd-YAG laser. The measurement range was 72–1560 cm^−1^.

The thermal properties of HNTs were investigated in the air using an STD 2960 thermogravimetry/differential thermal analysis (TA Instruments Inc., New Castle, DE, USA) device. The heating rate was 10 °C/min, and the temperature range was from room temperature to 800 °C.

A Brookhaven ZETAPALS device (Bookhaven Instruments, Holtsville, NY, USA) was utilized for measuring zeta potential values of halloysite nanofluids. The zeta potential (ζ) was determined from the electrophoretic mobility of HNTs utilizing the Henry equation by considering the Smoluchowski estimation [44]. Three repetitions of each sample were measured, then their average value was taken into consideration.

The rheological behavior of halloysite nanofluid was studied utilizing an Anton Paar Physica MCR 301 (Anton Paar, Ashland, VA, USA) rotation viscometer at various temperatures and shear rates. The number of shear rates per measurement was 10. The angular frequency range was 100 to 2000 s^−1^, while the amplitude was 5%.

The thermal conductivity of halloysite nanofluids was obtained utilizing an SKZ1061C TPS Thermal Conductivity Tester (SKZ Industrial, Shandong, China), which is based on the modified transient plane source approach. All samples were measured at five different temperatures of 20, 30, 40, 50, and 60 °C. A temperature-controlled oven was utilized to keep up the temperature at the defined setpoint.

## 3. Results and Discussion

### 3.1. Halloysite Structure

**Figure 1:**
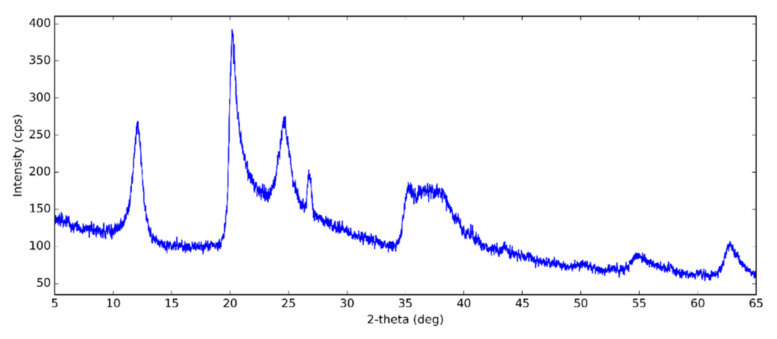
X-ray diffraction (XRD) pattern of halloysite at the following XRD conditions: X-Ray: 40 kV, 30 mA. Scan speed: 3.0 degree/min.

Figure 1 shows the XRD pattern of halloysite. The XRD pattern of the halloysite showed distinct diffraction peaks due to the crystalline property of the HNTs. This XRD pattern was indexed to ICDD (International Centre for Diffraction Data) 00-029-1487. The pattern had peaks corresponding to the metahalloysite or aluminum silicate hydroxide. The diffraction peaks at 2θ = 12.0, 20.1, 24.6, 35.0, 37.9, 54.5 and 62.6 corresponded to (001), (100), (002), (110), (003), (210) and (300) planes, respectively [45]. The presence of the (001) peak at 2θ of 12.0° corresponded to a layer spacing of 0.73 nm. This can be ascribed to halloysite-7 angstrom. The dehydrated state was also confirmed by the (100) diffraction peak at 2θ of 20.1° (0.44 nm). The layer distance of the hydrated halloysite is 10 Angstroms. After dehydration—which is an irreversible process—the layer distance collapses to 7 angstroms. This is characteristic of tubular halloysite [46,47].

**Figure 2:**
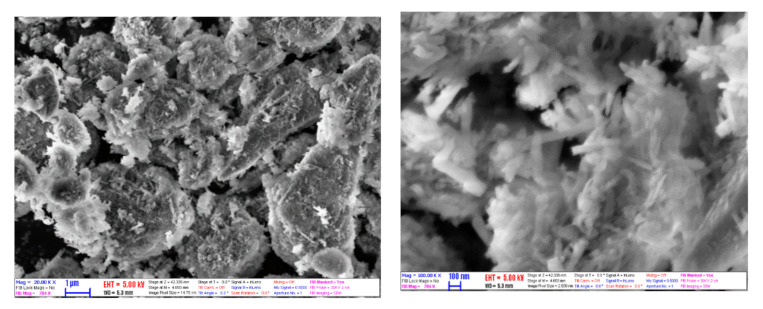
Scanning electron microscope (SEM) images of halloysite nanotubes (HNTs).

Figure 2 shows the SEM image of the morphological structure of HNTs. From SEM studies, it can be seen that the sample used was uniform in content, containing nanotubes with infrequent particle agglomerates. Between HNTs, some platy particles were presented due to residual kaolinite. By treating the image of micrographs, the mean outer diameter and the mean length were determined. The diameter and length were 58 and 436 nm, respectively. The aspect ratio was calculated as ca. 7.5. Compared to the HNTs used by Alberola et al. [30], the halloysite in this study is smaller. This can give the advantage of higher thermal conductivity and greater stability.

**Figure 3:**
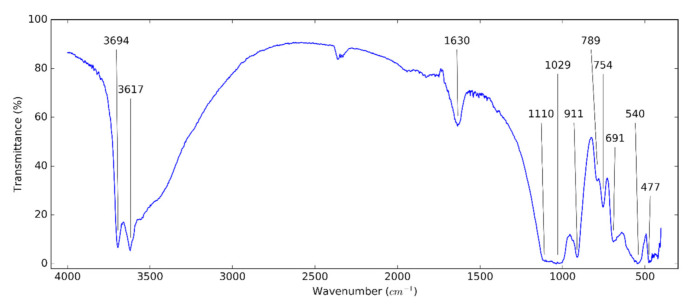
Fourier transform infrared (FT-IR) spectrum of HNTs.

The examination of functional groups of the used nanomaterials supports the effort to make a proper dispersion. Figure 3 shows the FT-IR spectrum of HNTs. The two infrared active modes centered at around 3694 and 3617 cm^−1^ are assigned to the stretching vibration (O–H) bonds of halloysite [48,49,50,51]. The peak at around 1630 cm^−1^ confirms the typical bending vibration of absorbed water. This peak in halloysite is more intense and broader than in kaolinite [52]. The peak at 1110 cm^−1^ is caused by the stretching mode of apical Si–O bonds. The peaks at 1029 and 477 cm^−1^ are attributed to the Si–O–Si asymmetric stretching and bending vibrations. The peaks at 911 and 540 cm^−1^ refer to the bending vibration of Al–O–H and Si–O–Al bonds, respectively. The peaks at 789 and 754 cm^−1^ are assigned to OH translation vibrations of HNTs [30,50]. The bands at 691 cm^-1^ are caused by the stretching vibration of apical O–H [49,53].

**Figure 4:**
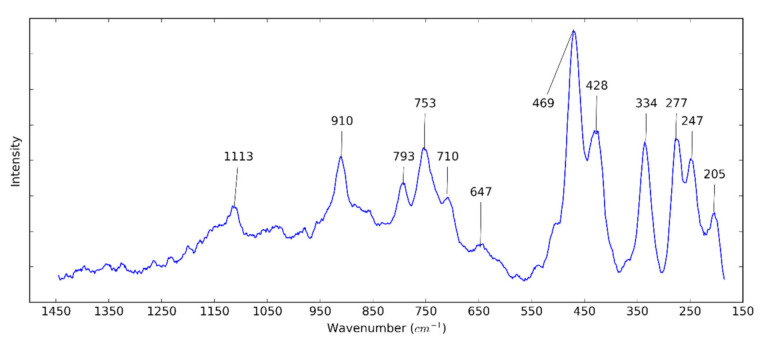
Raman spectrum of HNTs.

In Figure 4, the Raman spectrum of HNTs can be observed. The peak centered at 205 cm^−1^ refers to AlO_6_ octahedron (A_1g_). The peaks at 247 (A_1_) and 277 cm^−1^ (B_2_) are attributed to the internal vibrations of the O–H–O triangle. The three vibrational modes of the SiO_4_ tetrahedra are presented at 334, 428, and 469 cm^−1^. The peaks at 647, 710, and 747 cm^−1^ are caused by Si–O–Al translation. The peaks at 793 and 910 are assigned to the OH translation and liberation, respectively [54]. The peak at 1113 cm^−1^ (A_1_) confirms the stretching vibrations of the Si–O bond [55].

EDX provides the qualitative composition of the used material. The main components of HNTs, including Al, Si, and O (H can not be seen), are expected to see. Table 2 contains the EDX results of HNTs. The values in the table are the average of the results obtained at different measurement points in the atomic percentage. These values (Al:Si:O = 1:1.03:4.38) are similar to the EDX results (Al:Si:O = 1:1.13:5.42) obtained from Tayser et al. [53]. The oxygen content indicates that the used halloysite does not have H_2_O molecule.

**Figure 5:**
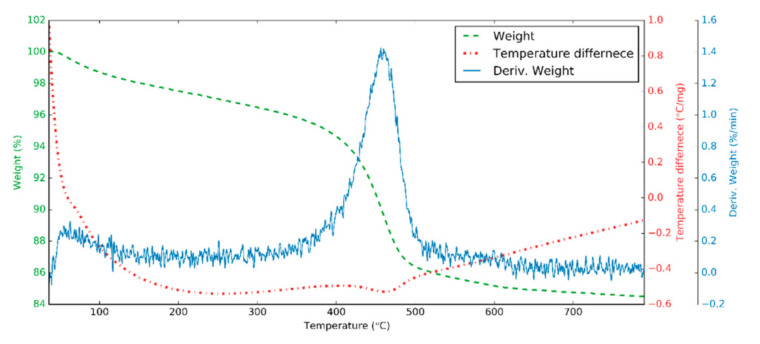
Thermogravimetry/different thermal analysis curve for HNTs.

Figure 5 shows the thermal analysis of HNT samples. At around 150 °C and below, weight losses refer to the loss of absorbed water (2%) on surface and interlayer [49]. From 150 °C to 400 °C, the interlayer water is removed completely [56], while at 400–500 °C, the Al–OH groups of HNTs are dehydroxylated with a loss of approximately 9% and the “metahalloysite” (Al_2_O_3_·2SiO_2_) is formed [49]. Above 500 °C, alumina-rich phase and amorphous SiO_2_ is formed distinctly. In the differential thermal analysis diagram, because of the removal of water, the process is endothermic, and then the structural rearrangement of the material—above 800 °C—is exothermic [57].

### 3.2. Zeta Potential Measurement

The zeta potential of 0.5% HNT nanofluids with different surfactants is shown in Table 3. Due to the improvement of the stability of HNT nanofluids, different surfactants are utilized, such as cetyltrimethylammonium bromide (CTAB), sodium dodecylbenzenesulfonate (SDBS), gum Arabic (GA), SCMC, oleylamine, and Triton X-100 (TX). Among the used surfactants, the SCMC gives the best result with −30.54 mV. According to the zeta potential values, the HNT nanofluids have acceptable stability with SDBS and SCMC. By visual observation, it can be confirmed that the SCMC is the best choice.

Colloidal solutions with zeta potentials as low as –30 mV have acceptable stability [6,58]. Table 4 shows the zeta potential values of HNT nanofluids with different concentrations. With surfactants, the zeta potential of 0.5, 1.0 and 1.5 vol% HNT nanofluids was −30.42, −33.03 and −43.33 mV, respectively, while with pH = 12 medium, the zeta potentials are −33.40, −39.72 and −32.39 mV on the same order. These values confirm the stability of nanofluids. Also, visual observation verifies that these nanofluids are stable for several days.

### 3.3. Rheological Properties of Halloysite Nanofluid

**Figure 6:**
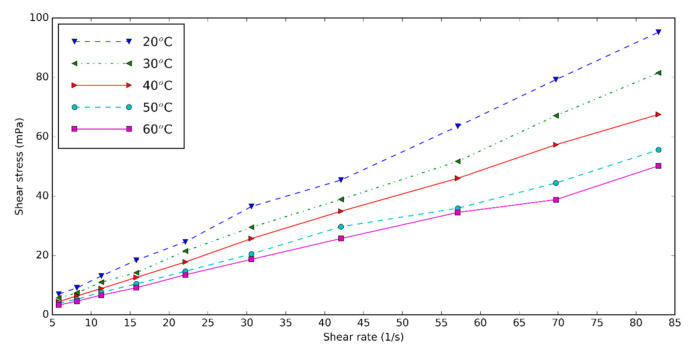
Shear stress–shear rates diagram of nanofluids for concentration of 0.5%.

The rheology and viscosity of the nanofluids are important parameters determining the heat transfer. The viscosity of the base fluid and HNT nanofluids at different shear rates is measured for three volume concentrations of 0.5, 1.0, and 1.5 at five temperatures: 20, 30, 40, 50, and 60 °C. Figure 6 shows the shear rate–shear stress diagram of 0.5 vol% HNT nanofluids with surfactant at different temperatures. Shear stress of HNT nanofluids falls with increasing temperature and rises with increasing concentration of nanofluids. The increase in temperature causes the Brownian movement and thermal motion of molecules to be higher, thus the viscosity of nanofluids decreases [59,60]. The shear rate of HNT nanofluid is almost linearly dependent on the shear rate. We conclude that the nanofluids are Newtonian.

**Figure 7:**
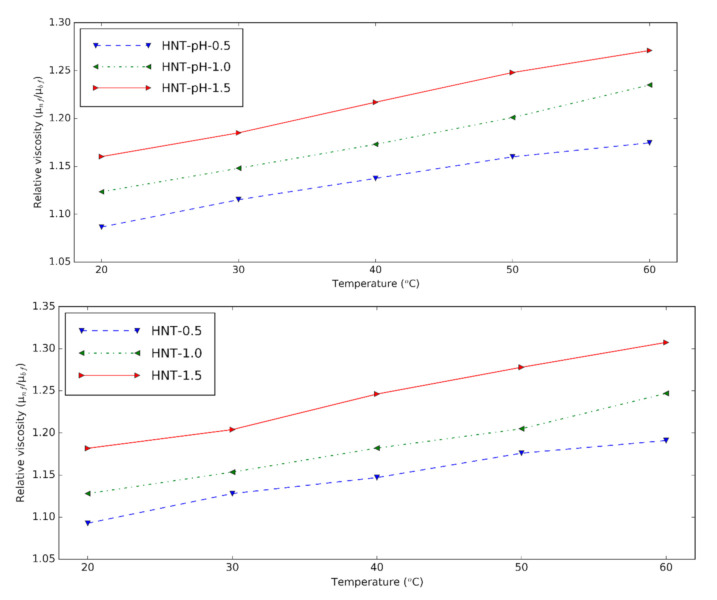
Relative viscosity of HNT nanofluids at different temperatures (upper: changing pH, lower: using surfactant).

Figure 7 shows the viscosity increment of prepared HNT nanofluids at different temperatures. Relative viscosity is obtained by dividing the viscosity of nanofluids by that of the base fluids. It can be seen that the viscosity is higher with nanoparticle content due to the clusters formed from nanoparticles [6]. Temperature plays an essential role in the relative viscosity. This means that temperature decreases the viscosity of base fluids more than that of the nanofluids. Compared to the HNT nanofluids at pH = 12, the relative viscosity of nanofluids containing the surfactant doesn’t have a significant difference. With surfactant, the HNT nanofluids have the lowest relative viscosity of 1.09 for 0.5% volume concentration and the highest relative viscosity of 1.31 for 1.5 vol%. The viscosity of nanofluids increased from 9% to 31% compared to the base fluid containing the surfactants.

### 3.4. Thermal Conductivity of Halloysite Nanofluid

**Figure 8:**
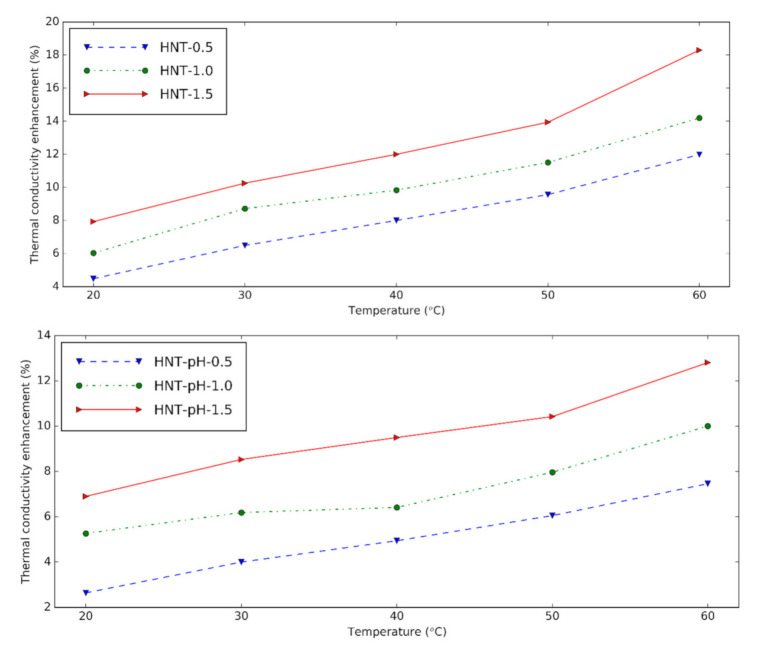
Thermal conductivity and enhancement of thermal conductivity of halloysite nanofluids at different temperatures (upper: changing pH, lower: using surfactant).

The thermal conductivity of HNT nanofluids at different temperatures is presented in Figure 8. The device is reliable within 0.6% error when the calibration measurement is verified for distilled water. The nanofluids show greater thermal conductivity than the base fluid at experimental temperatures. HNT nanofluids with surfactant give 4.48%, 6.03% and 7.93% thermal conductivity increment at 0.5 vol%, 1.0 vol%, and 1.5 vol% in comparison with the base fluid at 20 °C, respectively. By increasing the temperature, the thermal conductivity of nanofluids increases due to the augmentation in the Brownian motion of the solid.

It can be seen that when the nanoparticle content in nanofluids increases, the thermal conductivity also increases because of the higher number of nanoparticles presented in the nanofluid. The thermal conductivity enhancement of nanofluids containing surfactant is slightly higher than nanofluids with pH = 12. It is concluded that like changing pH, surfactant supports using HNT in the preparation of nanofluids. Compared to the results from Alberola et al. [30], the thermal conductivity of the nanofluids in this study is greater. This may be due to the smaller halloysite used in this research.

### 3.5. Regression Correlations

According to the results of Azmi et al. [61], the following correlations are proposed from the measured results:

(1) $$\mathrm{Relative}\;\mathrm{viscosity}=\frac{\mu_{\mathrm{nf}}}{\mu_{\mathrm{bf}}}=0.9775{\left(1+\frac{T}{70}\right)}^{0.26087}{\left(1+\frac{\phi }{100}\right)}^{8.13792}$$

(2) $$k_{\mathrm{nf}}=k_{\mathrm{bf}}\times 0.9777{\left(1+\frac{T}{70}\right)}^{0.18558}{\left(1+\frac{\phi }{100}\right)}^{3.64544}$$

where μ and k represent the viscosity and thermal conductivity; φ and T are volume concentration and temperature.

**Figure 9:**
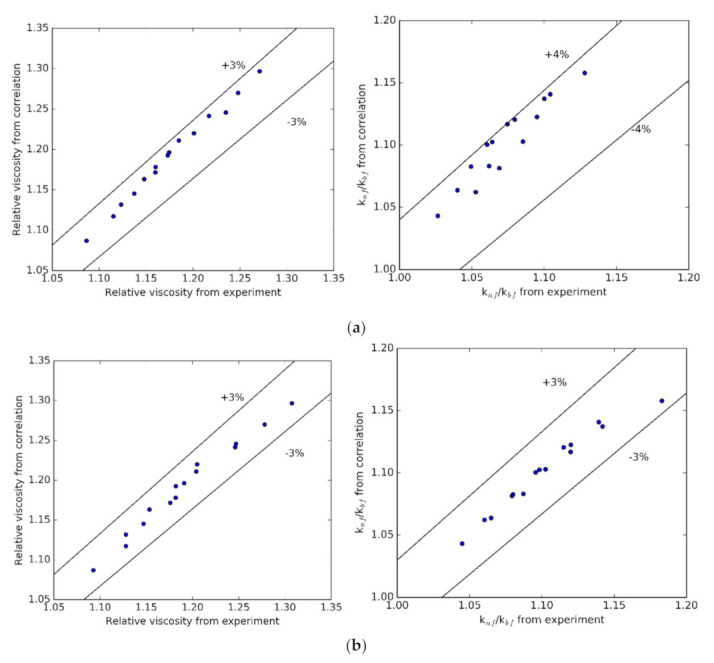
Comparison between thermal conductivity obtained from experiment and proposed correlation: (**a**) pH = 12, (**b**) surfactant.

The tabulation of viscosity and thermal conductivity from the experiment and the proposed correlations is shown in Figure 9a with pH = 12 and Figure 9b with the surfactant. The average and standard deviations are 0.68% and 0.86% for viscosity; and 0.43% and 0.74% for thermal conductivity. It is concluded that the proposed correlations are suitable for the experimental results.

## 4. Conclusions

In this research, HNT nanofluids were investigated at different concentrations and temperatures by using surfactants and changing pH. The results show the high purity, shape, and dimensions of the used HNT. The zeta potential measurements and visual observation proved the stability of the HNT nanofluids.

With surfactants, the HNT nanofluids have the highest thermal conductivity increment of 18.30% for 1.5 vol% concentration in comparison with the base fluid. The thermal conductivity enhancement of nanofluids containing surfactant is slightly higher than nanofluids with pH = 12. From the rheological measurements, it is shown that the nanofluids were Newtonian. The viscosity enhancements of the nanofluid were 11% and 12.8% at 30 °C for 0.5% volume concentration with surfactants and pH = 12, respectively. Instead of changing pH, the surfactants give good results for the preparation of the nanofluid. Novel equations of viscosity and thermal conductivity for these nanofluids were proposed.

## Figures and Tables

**Table 1:** Specification of halloysite nanofluid samples.

Sample Names	Halloysite (vol%)	DI (vol%)	1M NaOH Solution (vol%)
HNT-0.5	0.50	99.50	0.00
HNT-1.0	1.00	99.00	0.00
HNT-1.5	1.50	98.50	0.00
HNT-pH-0.5	0.50	98.50	1.00
HNT-pH-1.0	1.00	98.00	1.00
HNT-pH-1.5	1.50	97.50	1.00

**Table 2:** Energy-dispersive X-ray (EDX) analysis results of HNTs.

Element	Atomic%		
	Al	Si	O
Present work	15.59	16.13	68.28
Tayser et al. [53]	13.24	15.00	71.76

**Table 3:** Zeta potential of 0.5 vol% HNT nanofluids with different surfactants.

Surfactant	Zeta Potential of 0.5% HNT Nanofluid (mV)
N/A	−11.83
Tween	7.91
Oleylamine	24.24
CTAB	20.42
SDBS	−26.76
GA	−16.99
SCMC	−30.54

**Table 4:** Zeta potential of halloysite nanofluids with different concentrations.

Nanofluids	Zeta Potential (mV)
SCMC-0.5	−30.54
SCMC-1.0	−32.18
SCMC-1.5	−31.22
pH12-0.5	−33.40
pH12-1.0	−39.72
pH12-1.5	−32.39
